# Evaluation of Proinflammatory Chemokines in HIV Patients with Asymptomatic *Leishmania Infantum* Infection

**DOI:** 10.3390/tropicalmed8110495

**Published:** 2023-11-09

**Authors:** Bruna Eduarda Freitas Monteiro, Elis Dionísio da Silva, Gilberto Silva Nunes Bezerra, Marton Kaique de Andrade Cavalcante, Valéria Rêgo Alves Pereira, Maria Carolina Accioly Brelaz Castro, Luiz Gustavo Mendes, Diego Lins Guedes, Walter Lins Barbosa Júnior, Zulma Maria de Medeiros

**Affiliations:** 1 Graduate Program in Health Biosciences and Biotechnology, Aggeu Magalhães Institute, Oswaldo Cruz Foundation (Fiocruz), Recife 50670-420, PE, Brazil; valeria.hernandes@fiocruz.br; 2 Health and Biotechnology Institute, Federal University of Amazonas, Coari 69460-000, AM, Brazil; elisdionisio@ufam.edu.br; 3 Department of Nursing & Healthcare, Technological University of the Shannon: Midlands Midwest, N37 HD68 Athlone, Ireland; gilberto.bezerra@tus.ie; 4 Department of Immunology, Aggeu Magalhães Institute, Oswaldo Cruz Foundation (Fiocruz), Recife 50670-420, PE, Brazil; martonc@aluno.fiocruz.br (M.K.d.A.C.); maria.bcastro@ufpe.br (M.C.A.B.C.); 5 Parasitology Laboratory, Academic Center of Vitória (CAV), Federal University of Pernambuco, Vitória de Santo Antão 55608-680, PE, Brazil; 6 National Health Foundation, Petrolina 56304-020, PE, Brazil; 7 Medical School, Life Sciences Center, Academic Center of Agreste, Federal University of Pernambuco, Caruaru 55014-900, PE, Brazil; diego.linsguedes@ufpe.br; 8 Faculty of Medical Sciences, University of Pernambuco, Recife 50100-130, PE, Brazil; 9 Department of Parasitology, Aggeu Magalhães Institute, Oswaldo Cruz Foundation (Fiocruz), Recife 50670-420, PE, Brazil; walter.lins@fiocruz.br; 10 Institute of Biological Sciences, University of Pernambuco, Recife 50100-130, PE, Brazil

**Keywords:** chemokine, visceral leishmaniasis-HIV, HIV, asymptomatic

## Abstract

Asymptomatic *Leishmania infantum*, when associated with HIV, can become severe and potentially fatal. In this co-infection, the worst prognosis may be influenced by the host’s immunological aspects, which are crucial in determining susceptibility. Chemokines play an important role in this process by influencing the cellular composition at affected sites and impacting the disease’s outcome. Therefore, the aim of this study was to evaluate proinflammatory chemokines in HIV patients with the asymptomatic *L. infantum* infection. In this cross-sectional study, the levels of CCL2, CCL5, CXCL8, MIG, and IP-10 were measured in 160 serum samples from co-infected patients (*n* = 53), patients with HIV (*n* = 90), and negative controls (*n* = 17). Quantification was determined by flow cytometry. The obtained data were statistically analyzed using the Kruskal–Wallis test, followed by the Dunn’s post-test and the Spearman’s correlation coefficient. Significance was set at *p* < 0.05. The chemokines CCL2, CCL5, MIG, and IP-10 exhibited higher levels in the HIV group compared to co-infection. However, the elevated levels of all these chemokines and their increased connectivity in co-infected patients appear to be important in identifying proinflammatory immune responses associated with the asymptomatic condition. Furthermore, a weak negative correlation was observed between higher levels of CXCL8 and lower viral loads in co-infected patients.

## 1. Introduction

Visceral leishmaniasis (VL) is a serious parasitic disease caused by intracellular protozoa belonging to the *Leishmania donovani* complex, mainly affecting organs such as the liver, spleen, and bone marrow [1]. In the condition of VL with the human immunodeficiency virus (HIV), it represents a medical challenge in diagnosis and treatment due to the complex interactions between pathogens and changes in the immune system resulting from this co-infection [2]. Thus, the dysregulation of the immune system in HIV-infected individuals alters the immune response to the *Leishmania* spp. parasite, leading to severe complications associated with a high parasite load [3]. In turn, co-infection enhances viral infectivity and replication, further weakening the immune system [2]. As a result, it is associated with an increased risk of developing symptomatic VL and AIDS, along with a higher mortality rate compared to that observed in VL or HIV mono-infections [4,5,6].

*Leishmania* spp. and HIV synergistically promote increased host susceptibility to infections by impairing effector cell functions and, consequently, promoting a predominant Th2-type immune response (T helper 2). This response is characterized by the expression of anti-inflammatory molecules that compromise the body’s ability to control both infections, favoring severe manifestations of the diseases [7]. However, asymptomatic co-infected individuals have a predominant Th1 cellular immunity (T helper 1) that confers resistance to the development of co-infection. The expression of proinflammatory chemokines involved in this response plays a key role in protecting against infection by stimulating the activation and chemotaxis of effector cells, directing them to inflammatory sites [8].

Studies have reported that chemokine (C-C motif) ligand 2 (CCL2) stimulates the anti-*Leishmania* response in macrophages by mechanisms related to the expression of nitric oxide (NO) and reactive oxygen species (ROS) [9,10]. Together with chemokine ligand 5 (CCL5), it promotes granuloma formation and maturation in VL [11]. The chemokine (C-X-C motif) ligand 8 (CXCL8), in turn, participates in the activation and recruitment of neutrophils, which are important for the immune response against infection [12]. Monokine induced by IFN-γ (MIG) and IFN-γ-inducible protein 10 (IP-10) act on Th1 cell recruitment, macrophage leishmanicidal activity, and the upregulation of inflammatory mediators, resulting in intracellular parasite killing [13,14]. Furthermore, plasmatic levels of CCL2, CXCL8, MIG, and IP-10 were elevated in patients with VL without clinical signs and symptoms, identifying them as robust markers of asymptomatic infection and therapeutic efficacy [15,16,17,18,19].

Changes in the levels of these chemokines during HIV infection have also been previously described [2,20,21,22,23,24]. High levels of CCL2 can assist viral infection by recruiting target cells to affected sites and increasing the expression of CXCR4 (a co-receptor for HIV) [20]. Conversely, the elevated expression of CCL5 by activated natural killer (NK) cells suppresses HIV replication in vitro by interfering with the virus’s ability to utilize CCR5 as an entry co-receptor in CD4+ cells [21,22]. Increased levels of CXCL8, as well as CCL5, have also been associated with an in vitro reduction of viral load through transcriptional mechanisms and modulation of the CCR5 receptor [23,24]. Furthermore, it has been observed that the chemokines MIG and IP-10 are present at elevated levels in asymptomatic co-infection, in response to the increased expression of interferon-gamma (IFN-γ) [19], which has been shown to be negatively regulated by HIV in vitro [2].

However, in the context of co-infection, there are few studies that evaluate immunological molecules associated with susceptibility, which could provide relevant information for more appropriate clinical management [7]. Therefore, the present study aimed to assess the influence of an asymptomatic *L. infantum* infection on the levels of proinflammatory chemokines CCL2, CCL5, CXCL8, MIG, and IP-10 in HIV-infected patients.

## 2. Materials and Methods

### 2.1. Study Design and Samples

This was a cross-sectional study conducted with 143 HIV-positive patients confirmed through two rapid tests: rapid test 1 (RT1)—Núcleo de Doenças Infecciosas (Rapid Check HIV)—and rapid test 2 (RT2)—Bioeasy test (Standard Diagnostic Inc.) or Dual Path Platform (DPP)/HIV test (Biomanguinhos) [25]. The patients were aged 18 years or older and were receiving regular high-impact antiretroviral therapy (HAART) at the outpatient clinic of the Pernambuco Unified Health System (SUS), a VL-endemic area in Northeast Brazil.

All HIV patients without signs and symptoms of VL were screened for *L. infantum* in order to identify the co-infection. Following the recommendations of the The Ministry of Health of Brazil [26], we considered at least one positive result in diagnostic tests for VL: polymerase chain reaction (PCR) in peripheral blood, as described by Gualda et al. [27]; three serological tests using serum, including the Direct Agglutination Test (DAT) (Biomedical Research, Amsterdam, The Netherlands), Rapid Test rK39 (InBios International, Seattle, WA, USA), and Enzyme Linked Immunosorbent Assay (ELISA) [28]; and the Katex *Leishmania* antigen test in urine (Kalon Biological Ltd., Guildford, UK). In this way, we identified 53 co-infected patients. At the time of co-infection diagnosis, attending physicians followed the recommendation of the World Health Organization (WHO) [29] and the Pan American Health Organization (PAHO) [30] of not treating asymptomatic cases.

As a reference control group, 17 healthy individuals 18 years or older, living in Recife, Pernambuco, who voluntarily participated in the study, were included. They were only tested for VL, and the results were negative for the disease.

All samples were stored at −80 °C in the Laboratório de Doenças Transmissíveis do Instituto Aggeu Magalhães (IAM-Fiocruz) until used.

### 2.2. Data Collection and Laboratory Procedures

The following demographic, clinical, and laboratory data were collected through a questionnaire and medical records: gender, age, income (per month), irregular fever, hepatomegaly, splenomegaly, weight loss, cough, diarrhea, asthenia, pale mucous membranes, hemoglobin count, leucocytes, neutrophils, lymphocytes, T CD4+ e T CD8+ count, platelets, and HIV viral load.

CCL2, CCL5, CXCL8, MIG, and IP-10 chemokines were quantified in serum samples using the Cytometric Bead Array (CBA) system of the BD Human Chemokine kit (BD Biosciences, San Jose, CA, USA) in accordance with the manufacturer’s recommendations. Briefly, 50 μL of serum from each subject was incubated with 50 µL of capture beads (anti-CCL2, CCL5, CXCL8, MIG, and IP-10) and 50 µL of the Human Chemokine PE Detection Reagent for 3 h at room temperature, protected from light. Following incubation, 1 mL of Wash Buffer was added to each assay tube, followed by centrifugation at 200 g for 5 min. After removing the supernatant, 300 µL of Wash Buffer was added to resuspend the bead pellet. The limits of detection of these chemokines, as per the manufacturer, are as follows: CCL2—2.7 pg/mL; CCL5—1.0 pg/mL; CXCL8—0.2 pg/mL; MIG—2.5 pg/mL; IP-10—2.8 pg/mL. Data were acquired using a FACSCalibur flow cytometer (BD, San Jose, CA, USA) and subsequently analyzed in FCAP Array Software (version 3.0, BD, San Jose, CA, USA). Standard curves were derived from chemokines standards and chemokine levels were expressed as pg/mL.

### 2.3. Statistical Analysis

Differences in the proportions of categorical variables by group were compared using the chi-square test. To compare continuous variables, the t-Student or Mann–Whitney test was used, as appropriate. Multiple comparative analyses among three groups were conducted using the Kruskal–Wallis test, followed by the Dunn’s post-test. Spearman’s correlation coefficient was calculated to assess the correlation between chemokine levels and laboratory parameters. The data obtained were analyzed using GraphPad Prism Software (version 8.0, San Diego, CA, USA). Differences were considered statistically significant at *p* < 0.05.

In the correlation analysis, we adopted the classification described by Dancey and Reidy [31], which categorizes the correlation coefficients as follows: <0.4 (weak magnitude correlation), 0.4 to 0.6 (moderate magnitude correlation), 0.7 to 0.9 (strong magnitude correlation), and 1.0 (perfect magnitude correlation). The creation of correlation networks based on the correlation data of chemokine measurements was performed using the Python libraries NetworkX [32] and Matplotlib [33]. In summary, through the NetworkX library, a correlation network was created in which each chemokine is represented as a node in the network. The edges between the nodes represent the correlations between the chemokines. Matplotlib library was used to generate graphical visualizations of the correlation networks. In this process, nodes were represented as points on the graph and edges between the nodes were according to the calculated correlations. To represent the strength of the correlations, the thickness of the edges was adjusted according to the correlation values, allowing for immediate visual interpretation.

### 2.4. Ethics Statement

All subjects were adults of both genders and provided written, informed consent. The study was approved by the research ethics committee of Instituto Aggeu Magalhães, Fiocruz Pernambuco (approval number 61218816.1.0000.5190).

## 3. Results

### 3.1. Demographic, Clinical, and Laboratory Features

The study population was composed mainly of men (62.2%), with a mean age of 35 years (range: 17–58 years), and with an income of less than USD 249.2/month (Supplementary Table S1). Co-infected patients did not show clinical signs and symptoms of VL. In relation to laboratory parameters, the values presented by the co-infection and HIV groups did not show significant differences (Table 1).

### 3.2. Determination of Serum Chemokine Levels

**Figure 1:**
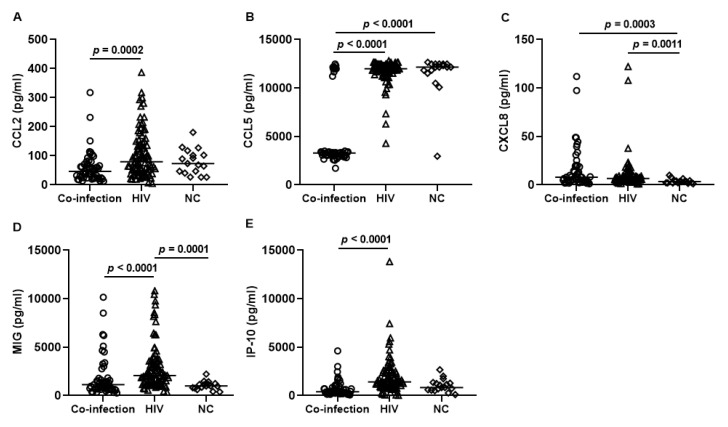
Comparison between serum chemokine levels in 53 co-infected patients, 90 HIV, and 17 negative control (NC). Kruskal–Wallis test, followed by the Dunn’s post-test; median and interquartile range. Concentrations (pg/mL) of (**A**) CCL2; (**B**) CCL5; (**C**) CXCL8; (**D**) MIG; (**E**) IP-10. Significant differences expressed with *p*-value.

The levels of the chemokines CCL2, CCL5, CXCL8, MIG, and IP-10 were compared among co-infected patients, HIV patients, and negative control individuals (NC), and the results are shown in Figure 1. Overall, the data analysis demonstrated that most chemokines (CCL2, CCL5, MIG, and IP-10) were elevated in HIV patients compared to co-infected (Figure 1A,B,D,E). On the other hand, lower levels of MIG and CXCL8 were observed among NC compared to the HIV group (Figure 1C,D), and higher levels of CCL5 in NC compared to the co-infection group (Figure 1B). In turn, higher levels of CXCL8 were observed in co-infected patients compared to the levels observed in the NC (Figure 1C). No significant differences were observed in serum CXCL8 levels between the two infection groups.

### 3.3. Correlation between Chemokine Levels and Laboratory Features

Our findings unveiled a weak negative correlation between the CXCL8 levels and viral load (r = −0.34) within the co-infection group (Table 2). In the HIV group, we observed a weak positive correlation between the CXCL8 levels and leukocyte counts (r = 0.25), and a correlation ranging from weak to moderate with neutrophils (r = 0.36) (Table 3). Regarding CCL2, the chemokine levels in the HIV group were weakly correlated with the white blood cell count (r = 0.23) and moderately correlated with neutrophils (r = 0.54). Furthermore, we observed weak negative correlations between the MIG levels and CD4+ T lymphocyte count (r = −0.21) in the HIV group.

**Figure 2:**
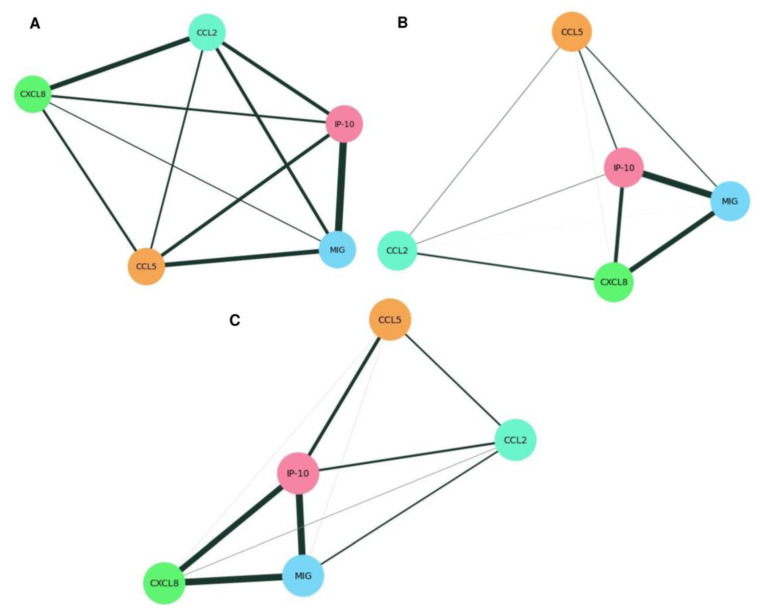
Correlation networks of serum chemokines between co-infection (**A**), HIV (**B**), and NC (**C**). The networks were constructed based on Spearman r scores between chemokines, with nodes representing the chemokines CCL2, CCL5, CXCL8, MIG, and IP-10, and edges representing the correlations between them. The edges were weighted based on the correlation values. Thus, weak correlations (r < 0.4) are represented by thin gray gradient lines and moderate correlations (r = 0.4 to 0.6) and strong correlations (r = 0.7 to 0.9) are represented by thick black lines between attribute pairs.

Furthermore, correlation analyses between chemokines in different groups (co-infection, HIV, and NC) revealed higher connectivity among the chemokines in the co-infection group (Figure 2A–C), with weak positive correlations between IP-10 and CCL2 (r = 0.29), IP-10, and CCL5 (r = 0.28), and correlations ranging from weak to moderate between MIG and CCL5 (r = 0.36), which were not observed in the other groups. Moderate positive correlations were also found between MIG and IP-10 (r = 0.58) and between CCL2 and CXCL8 (r = 0.40) in co-infected patients (Table 4).

In contrast to the co-infection group, we found a weak positive correlation between CCL2 and CXCL8 (r = 0.21) in the HIV group (Table 4, Figure 2B). Furthermore, we observed moderate positive correlations between the chemokines MIG and CXCL8 (r = 0.57) and between IP-10 and CXCL8 (r = 0.42), as well as a strong positive correlation between MIG and IP-10 (r = 0.75).

Similar to the HIV group, the NC group exhibited moderate positive correlations between IP-10 and CXCL8 (r = 0.62) and a strong positive correlation between MIG and IP-10 (r = 0.74). Additionally, a strong correlation between MIG and CXCL8 (r = 0.73) was identified for this group.

## 4. Discussion

An analysis of the serum levels of CCL2, CCL5, MIG, and IP-10 between co-infection and HIV patients highlighted higher levels of these chemokines in the HIV group. Furthermore, higher connectivity was observed among the chemokines, and a weak negative correlation between CXCL8 and the viral load in the co-infection group.

The HIV group exhibited higher levels of CCL2, with a positive association with leukocytes and neutrophils. The expression and release of chemokines in different cell types can be induced by oxidative stress and soluble factors associated with pathogens. In this context, it has been shown that HIV-derived Tat [34] and gp120 [35] proteins increase the expression and release of CCL2, which may facilitate the viral infection by upregulating the expression of CXCR4 (a co-receptor for HIV) and the recruitment of target cells to infection sites [20]. It has been demonstrated that the reduction of CCL2 levels was beneficial in the treatment of individuals with HIV [36], while high levels were associated with immunological failure in the viral infection [37].

However, we found that CCL2 levels were not associated with asymptomatic co-infection when compared with the other groups. As it performs antiparasitic actions in *L. infantum* and *L. donovani* by promoting the synthesis of NO and ROS in macrophages, chemokine expression can be influenced by the protozoan, favoring its replication [9,10]. Thus, high levels of the chemokine may hinder *Leishmania* spp. infection, and therefore, its increase has been linked to asymptomatic infection [16] and cure in cutaneous leishmaniasis [38]. Although the interaction between both pathogens plays an important role in the progression of both infections, there are still unknown mechanisms that may influence the pathogenicity of one or the other by increasing or decreasing CCL2 levels [7].

With regard to CCL5, we observed higher levels in the HIV group when compared to the co-infection group, which is consistent with the findings of Maksoud et al. [2]. They reported elevated levels of CCL5 in peripheral blood mononuclear cells (PBMC) infected with HIV compared to those co-infected with *L. donovani* and HIV. In Oliva et al.’s [21] study, in response to HIV infection, NK cells, when stimulated by IL-2, exhibited a significant mRNA expression of CCL5. According to these authors, the chemokine acts as a natural ligand for the CCR5 chemokine receptor (co-receptor for HIV), inhibiting viral replication.

Nevertheless, we found that CCL5 levels did not significantly differ between the HIV and negative control groups. The similarity in the high levels between these two groups in our study could explain the maintenance of a healthy state among HIV individuals, promoted by regular treatment with HAART. Although the co-infected individuals were also on antiretroviral treatment, the *Leishmania* spp. infection associated with the Th2 profile, which plays a modulatory role in the proinflammatory response, may have influenced the levels of this chemokine in this group.

It has been reported that in co-infection, the action of anti-inflammatory cytokines may influence the reduced expression of CCL5 and other molecules. According to Lazarski et al. [39], interleukin 4 (IL-4) attenuates the expression of the chemokines associated with the Th1 profile, such as CCL5, thereby limiting pathogen elimination. Additionally, a study conducted in the Northeast region of Brazil showed higher serum levels of IL-4 in the *L. infantum* infection in HIV patients [40].

In our study, the weak negative association between CXCL8 and the viral load identified in the co-infection may be related to the production of molecules in response to an *Leishmania* spp. infection, which modulate chemokine expression, thereby controlling the viral load. It has been shown that during symptomatic VL, the expression of interleukin-1beta (IL-1β), which promotes CXCL8 synthesis, is affected, leading to alterations in chemokine levels. These levels increase significantly after treatment [12,41]. Increased CXCL8 transcripts were also observed in keratinocytes activated in response to *L. infantum* [42]. Furthermore, elevated CXCL8 levels in asymptomatic individuals with VL [17] may reduce the viral load in infected cells through transcriptional mechanisms, similar to CCL5, by modulating the CCR5 receptor [23,24].

The chemokines MIG and IP-10 are primarily induced in response to stimulation by IFN-γ. Thus, the high levels of these chemokines observed in co-infected individuals in our study may be related to the previously documented increase of IFN-γ levels among asymptomatic HIV–*L. infantum* patients [19]. The elevated presence of IFN-γ, MIG, and IP-10 indicates the activation of the cellular immune response and the intensification of the inflammatory response, which have been suggested as biomarkers of an asymptomatic *Leishmania* spp. infection [15] and co-infection [19], thereby supporting our findings.

As this is a cross-sectional study involving asymptomatic patients, there is no information available regarding variations in the levels of these molecules in cases of clinical cure and symptomatic clinical evolution for VL. However, it was seen earlier that asymptomatic and cured individuals exhibit a heightened state of immune activation with elevated levels of proinflammatory chemokines compared to symptomatic individuals. According to Tasew et al. [43], there is a strong trend towards lower levels of the chemokines CCL2, CXCL8, and IP-10 in symptomatic VL.

Increased levels of anti-inflammatory cytokines (IL-10) and reduced proinflammatory cytokines (IL-2; tumor necrosis factor—TNF) were also observed in symptomatic VL–HIV, when compared to HIV and healthy individuals (*p* < 0.001) [44]. In turn, in our study, the verified levels of the chemokines CCL2, CCL5, CXCL8, MIG, and IP-10 in co-infected individuals may suggest an environment associated with a well-established immune response, as the study population consisted of asymptomatic individuals with a controlled viral load (<50 copies/mL) through regular HAART use. Furthermore, higher levels of CCL2, CCL5, MIG, and IP-10 in the HIV group, and greater connectivity among the chemokines in the co-infection group, suggest that the *Leishmania* spp. infection may influence the levels and activities of chemokines when associated with HIV. However, the elevated levels of all these chemokines in co-infected patients appear to be important in identifying the proinflammatory immune responses associated with the asymptomatic condition.

The monitoring of these chemokines could aid in preventing the development of VL and facilitating early disease management when it occurs alongside HIV. This is particularly valuable given the limitations of the commonly used conventional diagnostic tests [45]. Such an approach could prove especially beneficial in endemic areas for VL, particularly in Pernambuco, where there is a prevalence of 9.11% asymptomatic co-infection cases [46] and 16.9% symptomatic cases [47]. Our study represents a pioneering effort in evaluating these molecules in co-infection within Brazil.

In summary, the levels of chemokines and their correlations in our study indicate that these molecules play a collective role in the inflammatory immune response and control of the *L. infantum* infection associated with HIV. Therefore, we emphasize the importance of assessing these chemokines in co-infected individuals, considering that HIV significantly increases the risk of progression from asymptomatic *L. infantum* infection to clinical disease. The asymptomatic period provides a crucial opportunity for monitoring, through new strategies such as evaluating the chemokine profile, and intervention before this progression occurs. As future prospects, we intend to assess these molecules in VL and symptomatic VL–HIV in comparison to those in asymptomatic co-infected and HIV mono-infected patients. Furthermore, the assessment of a broader spectrum of cytokines and chemokines may provide a more comprehensive understanding of the immune response involved in co-infection.

## Figures and Tables

**Table 1:** Comparison of laboratory features between co-infected and HIV patients from Pernambuco, Brazil.

Laboratory Features	Co-Infection (*n* = 53)	HIV (*n* = 90)	*p*
Hemoglobin (g/dL)	13.39 ± 2.142 ^a^	13.87 ± 1.922 ^a^	0.20
Leucocytes (/mm^3^)	5212 ± 1850 ^a^	5817 ± 1587 ^a^	0.05
Neutrophils(/mm^3^)	2452 ± 1222 ^a^	3038 ± 1265 ^a^	0.06
Lymphocytes (/mm^3^)	1919 ± 892.9 ^a^	1985 ± 843 ^a^	0.75
Lymphocytes T CD4+ (/mm^3^)	623 (14–1795) ^b^	559.5 (12–1409) ^b^	0.89
Lymphocytes T CD8+ (/mm^3^)	867 (165–2779) ^b^	900 (332–3044) ^b^	0.19
Platelets (/mm^3^)	253,000 (74,000–487,000) ^b^	239,000 (125,000–636,000) ^b^	0.98
HIV viral load (copies/mL)			
Undetectable < 50	37 (70) ^c^	65 (72) ^c^	0.31
50–100.000	15 (29) ^c^	19 (21) ^c^	
>100.000	1 (1) ^c^	6 (7) ^c^	

^a^ Data presented as mean and standard deviation. ^b^ Data presented as median with the 25th and 75th percentiles in parentheses. ^c^ Frequency data (%).

**Table 2:** Correlation coefficients between serum chemokine levels and laboratory features of co-infected patients from Pernambuco, Brazil.

Co-Infection	CCL2	CCL5	CXCL8	MIG	IP-10
Hemoglobin (g/dL)	−0.08	−0.05	−0.22	−0.23	−0.17
Leucocytes (/mm^3^)	0.23	0.26	−0.09	−0.19	0.04
Neutrophils (/mm^3^)	0.25	0.04	0.06	−0.32	−0.05
Lymphocytes (/mm^3^)	0.16	0.08	−0.22	−0.39	−0.19
Lymphocytes T CD4+ (/mm^3^)	0.18	0.04	0.15	−0.20	−0.18
Lymphocytes T CD8+ (/mm^3^)	0.03	0.23	−0.03	0.08	0.05
Platelets (/mm^3^)	0.03	0.01	0.07	−0.18	−0.11
HIV viral load (copies/mL)	−0.05	−0.04	−0.34 ^a^	0.21	0.07

All values represent the Spearman r. ^a^ *p* < 0.05.

**Table 3:** Correlation coefficients between serum chemokine levels and laboratory features of HIV from Pernambuco, Brazil.

HIV	CCL2	CCL5	CXCL8	MIG	IP-10
Hemoglobin (g/dL)	0.13	0.13	−0.08	−0.14	−0.03
Leucocytes (/mm^3^)	0.23 ^a^	0.00	0.25 ^a^	−0.05	0.02
Neutrophils (/mm^3^)	0.54 ^b^	0.10	0.36 ^a^	0.15	0.06
Lymphocytes (/mm^3^)	0.17	0.02	−0.13	−0.20	−0.12
Lymphocytes T CD4+ (/mm^3^)	0.16	−0.17	−0.06	−0.21 ^a^	−0.16
Lymphocytes T CD8+ (/mm^3^)	0.11	−0.20	0.01	−0.08	−0.03
Platelets (/mm^3^)	−0.09	0.00	−0.06	−0.08	−0.12
HIV viral load (copies/mL)	−0.10	−0.09	0.02	0.12	0.01

All values represent the Spearman r. ^a^ *p* < 0.05; ^b^ *p* < 0.005.

**Table 4:** Correlation between CCL2, CCL5, CXCL8, MIG, and IP-10 serum chemokines in co-infection, HIV, and negative control (NC) from Pernambuco, Brazil.

Chemokine	CCL2	CCL5	CXCL8	MIG
Co-infection				
CCL5	0.15	-	-	-
CXCL8	0.40 ^b^	0.20	-	-
MIG	0.26	0.36 ^a^	0.09	-
IP-10	0.29 ^a^	0.28 ^a^	0.18	0.58 ^c^
HIV				
CCL5	0.06	-	-	-
CXCL8	0.21 ^a^	0.01	-	-
MIG	0.00	0.13	0.57 ^c^	-
IP-10	0.06	0.16	0.42 ^c^	0.75 ^c^
NC				
CCL5	0.19	-	-	-
CXCL8	−0.06	−0.01	-	-
MIG	0.17	0.01	0.73 ^b^	-
IP-10	0.24	0.37	0.62 ^a^	0.74 ^b^

All values represent the Spearman r. ^a^ *p* < 0.05; ^b^ *p* < 0.005; ^c^ *p* < 0.0005.

## Data Availability

All data supporting the study findings are included in this published article or in the Supplementary Material.

## Supplementary Materials

The following supporting information can be downloaded at: https://www.mdpi.com/article/10.3390/tropicalmed8110495/s1, Table S1: Comparison of demographic features between co-infected and HIV patients from Pernambuco, Brazil.
