# Supplementary material for: Evaluation of Proinflammatory Chemokines in HIV Patients with Asymptomatic Leishmania Infantum Infection
Source: Trop Med Infect Dis. 2023 Nov 9;8(11):495. doi: 10.3390/tropicalmed8110495 (PMC10675805; doi:10.3390/tropicalmed8110495)
Supplement: Supplementary file 1 [file tropicalmed-08-00495-s001.zip › tropicalmed-2561986-supplementary.pdf]

Table S1: Comparison of demographic features between co-infected and HIV patients from Pernambuco, Brazil.

**Demographic features**	**Co-Infection (*n* = 53)**	**HIV (*n* = 90)**	***p***
Gender			
Male	35 (66,04) [a]	54 (60) [a]	0.47
Female	18 (33,96) [a]	36 (40) [a]	
Age (years)	34 (18-57) [b]	35 (17-58) [b]	0.70
Income (month)			
< US$ 249.2	43 (81) [a]	65 (72) [a]	0.31
US$ 249.2-498.4	8 (15) [a]	23 (26) [a]	
> US$ 498.4	2 (4) [a]	2 (2) [a]	

[a] Frequency data (%). [b] Data presented as median with the 25th and 75th percentiles in parentheses.
